# Outcomes After Kidney injury in Surgery (OAKS): protocol for a multicentre, observational cohort study of acute kidney injury following major gastrointestinal and liver surgery

**DOI:** 10.1136/bmjopen-2015-009812

**Published:** 2016-01-14

**Authors:** STARSurg Collaborative

**Affiliations:** Academic Department of Surgery, Heritage Building, University of Birmingham, Birmingham, UK

## Abstract

**Introduction:**

Acute kidney injury (AKI) is associated with increased morbidity and mortality following cardiac surgery. Data focusing on the patterns of AKI following major gastrointestinal surgery could inform quality improvement projects and clinical trials, but there is a lack of reliable evidence. This multicentre study aims to determine the incidence and impact of AKI following major gastrointestinal and liver surgery.

**Methods and analysis:**

This prospective, collaborative, multicentre cohort study will include consecutive adults undergoing gastrointestinal resection, liver resection or reversal of ileostomy or colostomy. Open and laparoscopic procedures in elective and emergency patients will be included in the study. The primary end point will be the incidence of AKI within 7 days of surgery, identified using an adaptation of the National Algorithm for Detecting Acute Kidney Injury, which is based on the Kidney Disease Improving Global Outcomes (KDIGO) AKI guidelines. Secondary outcomes will include persistent renal dysfunction at discharge and 1 year postoperatively. The 30-day adverse event rate will be measured using the Clavien-Dindo scale. Data on factors that may predispose to the development of AKI will be collected to identify variables associated with AKI. Based on our previous collaborative studies, a minimum of 114 centres are expected to be recruited, contributing over 6500 patients in total.

**Ethics and dissemination:**

This study will be registered as clinical audit at each participating hospital. The protocol will be disseminated through local and national medical student networks in the UK and Ireland.

**Strengths and limitations of this study**

- Outcomes After Kidney injury in Surgery (OAKS) will be the first prospective study of postoperative acute kidney injury (AKI) in gastrointestinal and liver patients in the UK and Ireland. It will be disseminated through a collaborative medical student network, enabling rapid data collection on many thousands of postoperative patients. The results will be generalisable across the UK and Ireland.
- Unlike some previous studies that arbitrarily defined AKI, OAKS will identify AKI based on an algorithm in widespread routine clinical use in England. While, there are limitations to its application to patients with chronic kidney disease, the algorithm is based on the internationally recognised Kidney Disease Improving Global Outcomes (KDIGO) AKI staging guidelines.
- Although data will only be collected on variables for which there is a known biologically plausible causative relationship with AKI, this observational study can only identify associations (not causation) between possible predisposing factors and AKI. OAKS will be hypothesis generating, identifying areas for further study. It will also generate the data required to design and power robust randomised clinical trials in the future.
- The snap-shot audit methodology limits the quantity and complexity of data it is feasible to collect. Fluid therapy and sepsis may be key factors related to AKI, but it would be difficult to collect robust data on these variables in a student-driven audit. Data on intraoperative contamination will be collected as a surrogate for postoperative abdominal and wound sepsis. Anastomotic leak will be recorded as this is a significant early septic complication.
- Not all patients will have blood test results available during the 1 year follow-up window. It is possible that some patients will have persistent renal dysfunction at 1 year, but blood tests will be unavailable to identify this. Therefore, rates of persistent renal dysfunction identified in this study are likely to be under-estimates.

## Background

Studies in cardiac surgery suggest that postoperative acute kidney injury (AKI) is common and strongly associated with increased morbidity, mortality and healthcare costs,^1–3^ but there is less evidence on the incidence and burden of AKI following gastrointestinal and liver surgery. Previous studies have reported the incidence of postoperative AKI after gastrointestinal surgery between 1 and 22%;^4–7^ this broad range reflects both heterogeneity in definitions of AKI and the limitations of retrospective data analysis.

### The need for further evidence

The National Confidential Enquiry into Patient Outcome and Death ‘Adding Insult to Injury’ report recommended that predictable and avoidable AKI should never occur.^8^ Moreover, National Health Service (NHS) England has recognised AKI as a key priority for improvement in its 2015–2016 Commissioning for Quality and Innovation framework.^9^ However, there are currently no reliable estimates of the incidence of postoperative AKI in gastrointestinal surgery, making it impossible to benchmark and audit local performance. A lack of clarity regarding potential factors associated with the development of postoperative AKI makes it difficult to consistently identify targets for interventions aimed at reducing its incidence.^5^ ^7^ ^10^ Reliable data on patterns of postoperative AKI could inform future quality improvement projects and clinical trials.

### Primary aim

The primary aim of the Outcomes After Kidney injury in Surgery (OAKS) study is to determine the incidence of postoperative AKI after major gastrointestinal and liver surgery.

### Secondary aims

The secondary aims of the OAKS study are to determine the short-term and mid-term morbidity and mortality associated with postoperative AKI and to identify perioperative variables that are associated with postoperative AKI.

## Methods

### Study design

**Figure 1:**
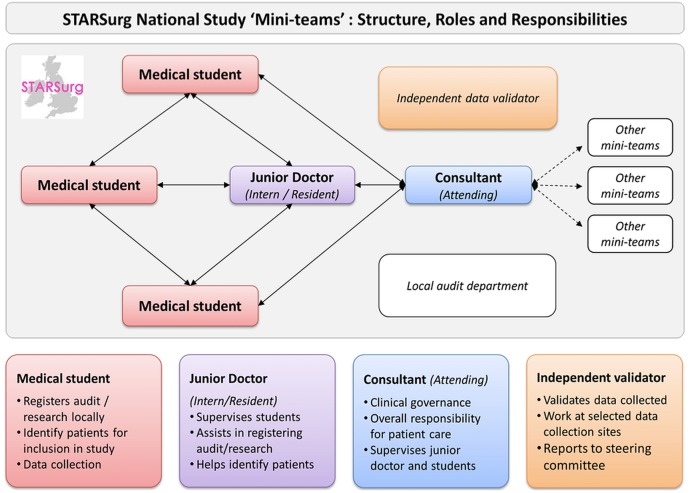
STARSurg ‘Mini-Team” structure, roles and responsibilities.

A multicentre prospective cohort study disseminated through collaborative medical student networks (figure 1).

### The STARSurg network

Student Audit and Research in Surgery (STARSurg) is the UK's national medical student research collaborative, run by the Surgical Research Gateway (SURG) Foundation (registered charity number 1 159 898). STARSurg is co-ordinated by a team of medical students and surgeons. The collaborative model and the educational benefits to participating students have previously been described elsewhere.^11^ ^12^

### Study setting

Hospitals in the UK and the Republic of Ireland that offer elective or emergency gastrointestinal or liver surgery will be invited to participate. Each centre may contribute up to four, 2-week sets of consecutive patient data.

### Inclusion criteria

- Adult patients (age ≥18 years) undergoing any gastrointestinal resection, liver resection or reversal of ileostomy or colostomy;
- Elective and emergency patients may be included;
- Open, laparoscopic, laparoscopic-assisted, laparoscopic-converted and robotic procedures may be included.

### Exclusion criteria

- No baseline preoperative creatinine available;
- Previous kidney transplant;
- Renal replacement therapy (any form of dialysis) in the 90 days preceding operation;
- Appendicectomy;
- Transplant surgery;
- Gynaecological primary indication;
- Urological primary indication;
- Vascular primary indication.

### Patient identification and data collection

Operating lists and theatre computer systems will be screened daily to identify eligible patients. The preoperative and operative data fields will be completed at the earliest opportunity following surgery. Collaborators will actively follow-up patients in the postoperative period, completing 30-day and 1 year follow-up promptly.

### Primary outcome

The primary outcome measure will be the incidence of AKI within 7 days of the indexed operative procedure. This period of 7 days has been selected to only capture AKI directly attributable to insults sustained during the perioperative period.

**Figure 2:**
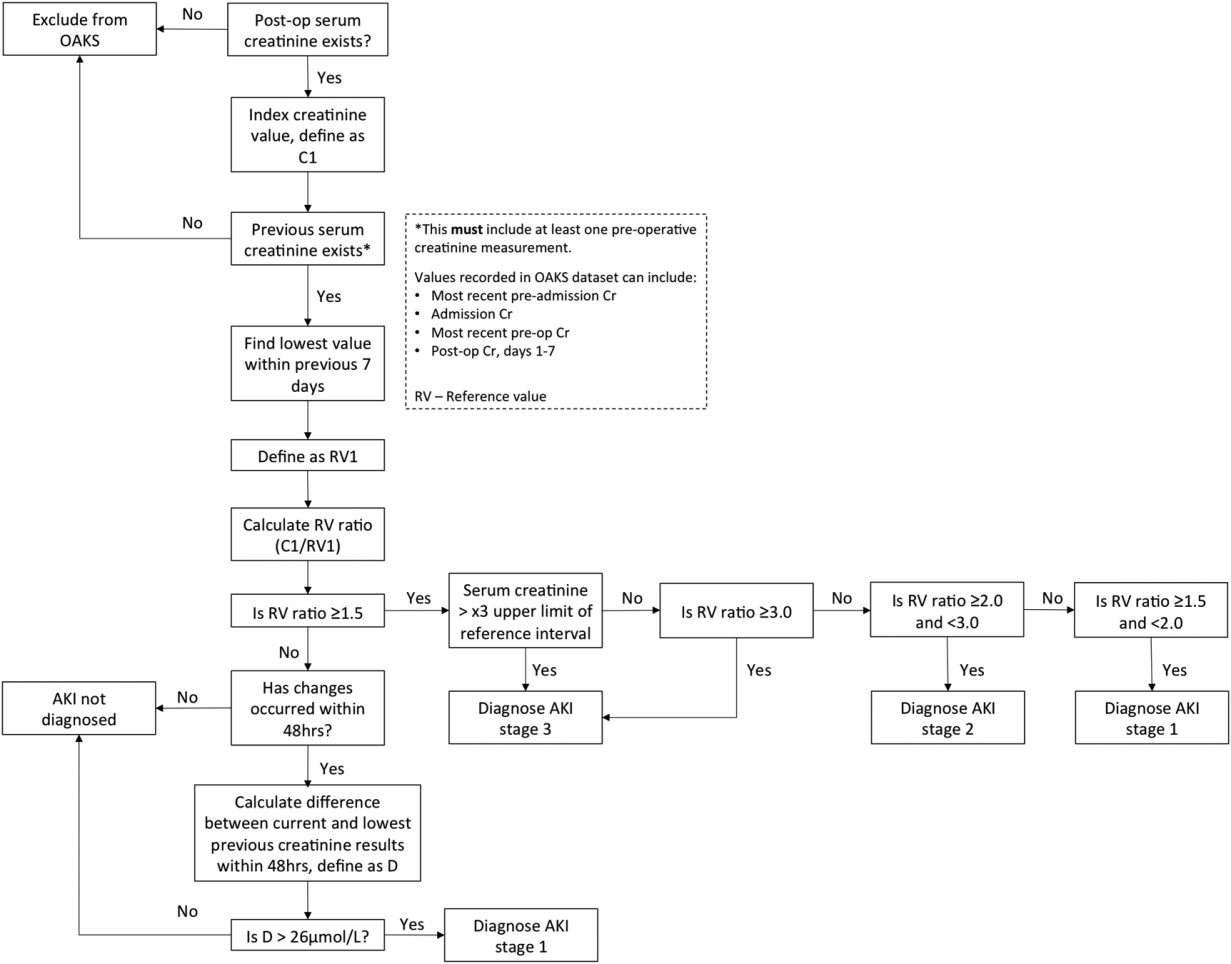
Adaptation of the national algorithm for detecting acute kidney injury.^14^ RV, reference value; OAKS, Outcomes After Kidney injury in Surgery.

Collaborators will submit raw creatinine values to the study database. AKI will be identified using an adaptation of the National Algorithm for Detecting Acute Kidney Injury classification system (figure 2), which is based on Kidney Disease Improving Global Outcomes (KDIGO) AKI guidelines.^13^ It was introduced by NHS England in June 2014 in a Patient Safety Alert requiring standardisation of the identification of AKI across the UK.^14^ If serum creatinine is not available over the past 7 days, the National Algorithm defines baseline creatinine as the median of all measured creatinine levels over the past year. It would not be practical for study collaborators to manually derive this value; therefore the algorithm has been adapted to require the most recent preoperative serum creatinine measurement. To identify emergency patients who had already developed AKI at admission, both the first creatinine measured on admission and the most recent preadmission creatinine will be required for emergency patients. A full description required creatinine data points is available in the online supplementary file (data points 11 and 29). In this observational study no extra blood tests will be performed, other than those ordered by the clinical team.

### Secondary outcomes

Secondary outcome measures will include:

- Incidence of AKI within 48 h of surgery
- Persistent renal dysfunction at discharge
- Persistent renal dysfunction at 12 months
- Regular renal replacement therapy (any form of dialysis) at 12 months
- 30-day major complication rate (Clavien-Dindo grades III-V)
- 30-day minor complication rate (Clavien-Dindo grades I-II)
- Mortality at 12 months

Persistent renal dysfunction will be defined as an increase from baseline of chronic kidney disease (CKD) stage, as defined by the Kidney Disease Outcomes Quality Initiative.^15^ In addition to serum creatinine, all other variables required to calculate estimated glomerular filtration rate (eGFR) using the Chronic Kidney Disease Epidemiology Collaboration formula will be collected.^16^ For the 30-day outcome, the single eGFR value nearest to postoperative day 30 will be used. For the 1 year outcome, blood tests taken 9–15 months postoperatively will be considered. Two separate eGFR values recorded at least 3 months apart will be required to demonstrate a persistent increase in CKD stage.

Complications will be recorded using the Clavien-Dindo classification, a standardised, internationally validated scoring system for postoperative complications.^17^ The highest grade complication (grade I-V) experienced by each patient will be recorded.

### Factors associated with AKI

A key aim of OAKS is to identify perioperative factors that may be associated with subsequent development of AKI. To record this, data will be collected on patient-related, disease-related and pharmaceutical variables (table 1). Detailed description of all data fields is available in the online supplementary file.

**Table 1:** Explanatory data variables collected for OAKS Study

Patient	Disease	Pharmaceutical
Age	Urgency of operation	Perioperative use of NSAIDs
Gender	Underlying pathology	Perioperative use of ACEi or ARBs
Ethnicity	Intraoperative contamination	Perioperative use of diuretics
American Society of Anaesthesiologists (ASA) Score	Baseline haemoglobin and albumin	Postoperative aminoglycosides
History of ischaemic heart disease	Perioperative red cell transfusion	Perioperative use of intravenous contrast
History of congestive heart disease		
History of cerebrovascular disease		
History of hypertension		
History of diabetes		
Smoking status		

ACEi, ACE inhibitors; ARB,angiotensin-receptor blocker; NSAIDs,non-steroidal anti-inflammatory drugs; OAKS, Outcomes After Kidney injury in Surgery.

### Centre questionnaire

Supervising consultants will be asked to complete a short centre questionnaire about the resources available at their centre (table 2).

**Table 2:** Centre questionnaire

Question	Options
(1) Are patients undergoing *elective* major surgery at your centre reviewed at a formal preoperative assessment clinic?	Yes—all patients; Yes—high-risk patients only; No
(2a) Do your centre's U&E (urea and electrolytes) results include AKI stage?	Yes; No
(2b) Does your centre have an electronic alerting system to flag patients that have developed AKI?	Yes; No
(3) Does your centre employ a full-time on-site nephrologist (consultant or associate specialist grade at this hospital)?	Yes; No
(4) Does your centre offer inpatient dialysis?	Dialysis in ICU only (CRRT); Acute dialysis in a renal centre; Both
(5) Is intraoperative goal directed fluid therapy available at your centre for major surgery?	Yes—routinely employed (>50% of cases); Yes—not routinely employed (<50% of cases); No
(6) Are patients undergoing major surgery at your centre admitted to high dependency/intensive care units postoperatively?	Yes—routinely (>50% of patients); Yes—most high-risk patients only; Never

AKI, acute kidney injury; CRRT, continuous renal replacement therapy; ICU, intensive care unit.

### Quality assurance

Each local team of medical students will be supervised by a qualified doctor (ranging from foundation year to registrar grade) and a consultant surgeon.

A detailed protocol for collaborators describing how to register and run the study will be made available online. It will provide a detailed data dictionary, along with guidance on potential data sources for collaborators to use when collecting the data. The protocol will be presented at collaborator meetings across the UK, enabling participants to clarify any uncertainties in person with the steering committee.

Collaborators will be asked to complete mandatory online e-learning modules covering study inclusion criteria, AKI, the Clavien-Dindo classification system and data governance protocols. This will ensure collaborators are able to safely collect accurate data.

To identify any local issues regarding data collection, all participating centres will be asked to pilot patient identification and case record completion for 1 day in the week leading up to the main study starting date. The steering committee will offer support throughout this period to resolve any problems.

### Validation

Only data sets with >95% data completeness will be accepted. Final year medical students or qualified doctors not involved in initial data collection will act as independent assessors, reviewing data collected at a local centre. Overall independent assessors will validate a minimum of 5% of patient records, with a target of >95% case ascertainment and >98% data accuracy.

### Data management

Data will be collected and stored online through a secure server running the Research Electronic Data Capture (REDCap)^18^ web application, hosted at the University of Edinburgh. REDCap is widely used internationally by academic organisations to store research databases. Collaborators will be given secure login details including a password for the REDCap project server. All transmission and storage of web-based information by this system is encrypted. Any patient identifiable information will not be available for data-analysis and will be automatically stripped from the database when exported from REDCap.

### Anticipated minimum recruitment

Based on our previous collaborative studies,^19^ an average centre performs approximately 20 operations meeting the study inclusion criteria in a 14-day period. At most centres in our previous study three data collection teams participated at each centre, collecting data over consecutive 14-day periods. Previously at least four centres participated at each of the 38 medical schools involved. Overall, we estimate that a minimum of 6840 patients will be recruited. The observational nature of the study's primary outcome means a power calculation is not required.

### Statistical analysis

Differences between demographic groups will be tested with the χ^2^ test for categorical data, with differences in continuous data analysed using Student t test. Two-sided statistical significance will be defined at the level of p<0.05. Multivariable binary logistic regression will be used to provide adjusted estimates of effects. Variables to be entered to the model will be those that occur prior to the operation. Initially all these variables will be considered. Variables to remain in the final model will be selected by best using Akaike Information Criterion (AIC). Effect estimates will be presented as ORs and bootstrapped 95% CIs and p values. All relevant second order interactions will be examined. We will explore the possibility of creating a risk prediction model. Such a model would be derived from one half of the data set and validated in the other half. Data handling and analysis will be performed in REDCap and R (R Foundation Statistical Programming, Vienna, Austria).

## Ethics and dissemination

### Research ethics approval

OAKS is a clinical audit based on National Institute for Health and Clinical Excellence (NICE) Clinical Guidelines requiring preoperative and postoperative measurement of serum creatinine in all patients undergoing major surgery.^20–22^ The South East Scotland Research Ethics Service advised that ethical approval is not required for OAKS in the UK. Participating centres will register OAKS locally as a clinical audit. They will also ensure they have successfully applied for Caldicott Guardian approval to submit anonymised patient data to a national audit and secure online database. In the Republic of Ireland collaborators are responsible for securing approval for the study from their local research ethics service.

### Protocol dissemination

The protocol will be disseminated primarily through the existing STARSurg network, medical student, surgical and medical societies, and postgraduate research collaboratives. A student local lead will be designated at each medical school to facilitate local study co-ordination. STARSurg has successfully developed a social media-based strategy for disseminating study protocols,^23^ including utilisation of Twitter (http://www.twitter.com/STARSurgUK) and Facebook (http://www.facebook.com/STARSurgUK).

## Discussion

This multicentre study will robustly measure the incidence and impact AKI in patients undergoing elective and emergency major gastrointestinal and liver surgery. By reliably determining the burden of AKI-associated morbidity and mortality, this study will define targets for future quality improvement programmes and clinical trials. Identifying risk factors for AKI will allow stratification of patients to prioritise future interventions aimed at enhancing preoperative optimisation and perioperative monitoring.

The study will be delivered by a national student collaborative network, pairing medical students with supervising doctors. The collaborative student-led and trainee-led collaborative groups have a track record in producing high-quality data sets.^19^ ^24^ A comprehensive study protocol, online training modules and consultant supervision will ensure consistent and reproducible data collection. Preplanned data validation will allow case ascertainment and data accuracy rates to be estimated.

The interpretation and comparison of previous studies exploring postoperative AKI has been challenging due to the lack of consistency in the definition of AKI. The NHS AKI Algorithm has standardised the recognition of AKI across England.^14^ Using an adaptation of this algorithm will ensure this study's findings are clinically applicable; the algorithm has been simplified to streamline data collection, but these changes are unlikely to alter the resultant AKI staging in a significant number of patients.

The greatest limitation of any observational study is, while able to identify associations between AKI and various risk factors, it will not be able to prove a definite causative relationship. Consequently, data will only be collected on variables for which there is a known biologically plausible causative relationship with AKI. The lack of reliable existing data in this field means that further observational data is required prior to the planning of any clinical trial that could produce higher grade evidence.

The snap-shot audit methodology which will be used by this study has previously been shown to collect accurate data.^19^ This methodology however limits the volume and complexity of data that can be collected. Perioperative fluid and blood loss have previously been associated with AKI.^10^ ^25^ The wide variation in fluid management across UK centres would make it difficult to capture meaningful data on fluid therapy within an observational, snapshot audit. Since blood loss is not universally accurately measured and recorded, the requirement for perioperative blood transfusion will be used as a surrogate for significant intraoperative blood loss. Sepsis may also predispose to AKI.^10^ ^26^ Although it may be difficult for this collaborative study to accurately recognise sepsis, the extent of intraoperative contamination will be used as a surrogate for risk of intra-abdominal and wound sepsis. In addition, data will be collected on anastomotic leak, as a key early complication that may drive sepsis in gastrointestinal patients.

Finally, this collaborative project will further strengthen and develop the STARSurg student collaborative network, encouraging medical students to participate in clinical academic studies while providing them with audit and research skills training.^11^
